# An Investigation of Heavy Metal Accumulation in Liver and Kidney Tissues of the European Hare (*Lepus europaeus* P.) in Samples Originating from Intensive Agricultural Environments in Hungary

**DOI:** 10.3390/toxics14080725

**Published:** 2026-08-16

**Authors:** Attila Bende, László Bánáti, László Könyves, Balázs Berlinger, István Fekete

**Affiliations:** 1 Institute of Wildlife Biology and Management, University of Sopron, Bajcsy-Zs. u. 4, 9400 Sopron, Hungary; banati.laszlo@phd.uni-sopron.hu; 2 Veterinary Centrum Kft., Balfi út 143, 9400 Sopron, Hungary; 3 Department of Animal Hygiene, Herd Health and Mobile Clinic, University of Veterinary Medicine Budapest, István u. 2, 1078 Budapest, Hungary; konyves.laszlo@univet.hu (L.K.); berlinger.balazs@univet.hu (B.B.); 4 Amity Institute of Psychology & Allied Sciences, Amity University Kolkata, Major Arterial Road, Action Area II, Rajarhat, New Town, Kolkata 700156, West Bengal, India; ifekete@kol.amity.edu

**Keywords:** herbivore mammal, European brown hare, heavy metals, toxicity, liver, kidney

## Abstract

The aim of this study was to evaluate the concentrations of 17 heavy metals measured in the liver and kidney tissues of the European brown hare (*Lepus europaeus*) as a function of age, sex, organ type, and sampling area. In addition, we assessed the relationships between trace element profiles and reproductive parameters and highlighted their potential relevance from a food-safety perspective. Age-related comparisons revealed significantly higher concentrations of Ti, Fe, Cd, and Hg in adult individuals, whereas no statistically significant differences were detected for the remaining elements. No significant sex-related differences were found for any of the investigated elements. In contrast, the organ-based analysis revealed substantial differences. The concentrations of all elements differed significantly between the liver and the kidney. Correlation analyses identified positive associations between Mn and Zn concentrations, as well as between Mn and Cu concentrations, in both organs. The relationship between Zn and Cd was also positive and statistically significant. A comparison among sampling areas showed no significant spatial differences in the concentrations of Cr, Fe, Cu, and Mo. The greatest spatial heterogeneity was observed for Co, Hg, Pb, and Se, with Se concentrations differing significantly among nearly all sampling areas. Our results indicate strongly overlapping spatial heavy metal profiles, suggesting a gradual rather than sharply differentiated geographical pattern and similar emission characteristics. This interpretation is further supported by the clustering and principal component analysis (PCA) results. PERMANOVA analyses indicated that trace element profiles did not significantly influence the number of placental scars. Furthermore, neither age nor its interaction with trace element profiles exerted a detectable effect on variation in placental scar numbers.

## 1. Introduction

The bioaccumulation of heavy metals in the biosphere is predominantly associated with anthropogenic processes, particularly, intensive environmental exploitation. In agricultural environments, the main emission sources include the use of pesticides and fertilizers and the combustion of fossil fuels [1,2,3,4,5]. Owing to population growth, industrialization, and the intensification of agricultural activities, these hazardous substances are increasingly accumulated in living organisms at elevated concentrations [6,7]. Numerous studies have demonstrated that heavy metal accumulation exerts detrimental effects on natural ecosystems [8,9,10,11], and their biomagnification also has pronounced negative impacts at higher trophic levels [12]. Although game meat is not among the most widely consumed types of meat in Hungary, it nevertheless plays a non-negligible role in human nutrition, as 61.0% of the population consumes game-derived products [13,14]. According to the most recent estimates, the average per capita consumption of game meat in Hungary is approximately 0.2 kg per year [15]. At the same time, more than 90% of the approximately 3800 tons of processed meat derived from 11,000 tons of harvested game (2019) is marketed in other EU Member States, amounting to nearly 3500 tons annually [14]. These figures indicate that game meat has a meaningful role in food supply chains. However, consumption of game meat—particularly offal—may represent a significant risk factor in terms of heavy metal exposure. Contrary to common perceptions, game meat is not necessarily healthier than meat from domesticated animals. Indeed, several studies have demonstrated that contaminant levels in wild animals may even exceed those found in livestock [12,16]. The accumulation of toxic heavy metals in soil and plants is therefore of critical importance, as herbivorous and omnivorous species can readily become contaminated through their diet, ultimately exposing humans to adverse health effects through biomagnification processes. Consequently, from both food-safety and public-health perspectives, these processes require detailed investigation [1,2,17]. Accordingly, increasing attention within ecotoxicological research has been directed toward huntable species inhabiting agroecosystems, particularly long-lived species that also have relevance for human consumption. Among these species, the European hare represents a potentially suitable bioindicator of heavy metal contamination in agricultural landscapes [18]. Through such species, current insights into environmental contamination in agroecosystems can be obtained [12], thereby providing a scientific basis for the development of conservation and management strategies aimed at protecting these biological communities [7].

Although the European hare is the most common small game mammal in Hungary, with significant importance in lowland hunting management [19], only limited literature data is available from the past decades, and it is based on small sample sizes and focuses on a restricted number of heavy metals [20,21]. Despite evidence from previous international studies indicating that environmental pollution directly affects hare survival and reproduction [22,23], and despite a significant population decline observed in Hungary over the past half-century [19,24,25], the underlying drivers remain insufficiently understood [25]. To evaluate the reproductive performance of the species in relation to heavy metal exposure, we used placental scar counts as our reproductive indicator. This parameter provides a reliable estimate of litter size when considered together with the number of parturitions and allows for comparison with previously published data on the reproductive performance of the European hare. Furthermore, we examined potential relationships between the concentrations of individual heavy metals and reproductive output across five sampling areas representing different regions of Hungary. To the best of our knowledge, no previous study has investigated this relationship in the European hare.

The present study aims to address the existing knowledge gap regarding heavy metal exposure in the European hare by analyzing organ samples from typical Hungarian hare habitats, covering 17 heavy metals, and considering implications for food safety.

## 2. Materials and Methods

In ecotoxicology, the two most frequently studied tissues—known as primary sites of substantial contaminant accumulation and responsible for detoxification processes [26]—are the liver and kidney. Accordingly, the accumulation of 17 heavy metals (Ti, V, Cr, Mn, Fe, Co, Ni, Cu, Zn, As, Se, Mo, Sb, Cd, Sn, Hg, Pb) was investigated in the European hare.

Since the animals were not hunted specifically for the purposes of this study, and as no tissue sampling from live animals was performed, the research conducted in this work does not fall under the scope of Government Decree 40/2013 (II. 14.) [27] on animal experiments. Therefore, no ethical approval procedure was required.

### 2.1. Sampling Area

**Figure 1:**
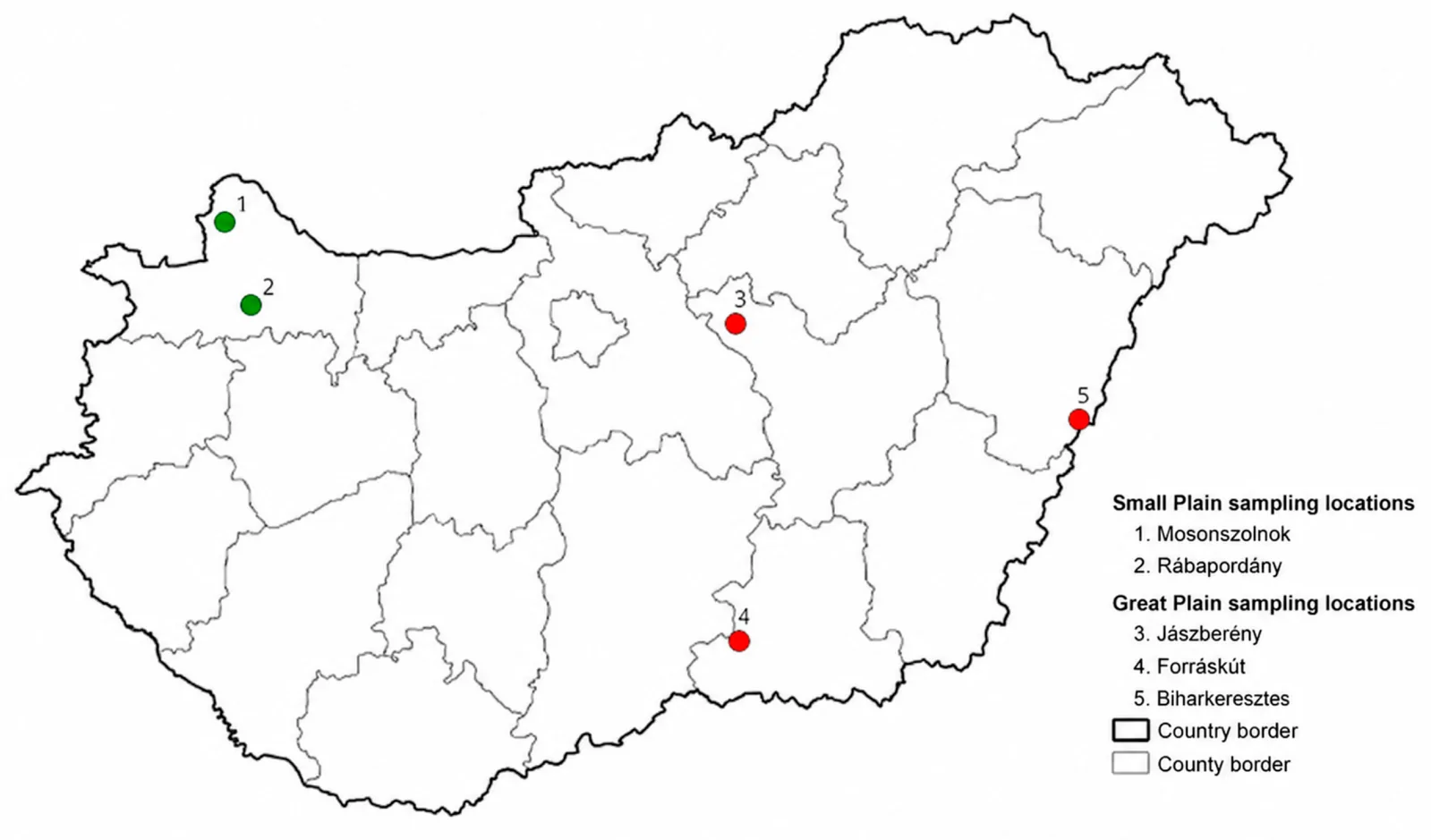
Sampling areas in the Little Hungarian Plain (Kisalföld) and the Great Hungarian Plain (Nagyalföld).

During the 2023 hunting season, a total of 163 shot European hares were collected, of which 157 kidney and 159 liver samples were suitable for laboratory analysis. The discrepancy resulted from organ damage during harvesting; consequently, 6 kidney and 4 liver samples were unsuitable for analysis. The hares were not harvested for the purposes of this study; rather, samples were randomly collected from hunting bags during the regular hunting season, and the sampled individuals were subsequently used for human consumption as game meat. The sampling sites were located in regions characterized by particularly high-quality European hare habitats, namely the Little Hungarian Plain and the Great Hungarian Plain. All sampling sites were located within intensively managed agricultural landscapes, typically characterized by large-scale fields exceeding 100 ha. Across all study areas, habitat heterogeneity was primarily provided by shelterbelts, tree lines, small patches of woodland and shrubs, and reed-fringed drainage channels. The selection of sampling sites was not based on presumed differences in environmental exposure to heavy metals. Instead, the sites were chosen to represent the characteristic habitats of the European hare in Hungary. Particular emphasis was placed on the Little Hungarian Plain and the Great Hungarian Plain, which support the highest hare densities and represent the species’ most suitable habitats. This sampling design enabled us to obtain a representative nationwide overview of trace element profiles. Consequently, this study provides a realistic representation of the agricultural environments that constitute the typical habitat of the European hare in Hungary. The geographic distribution of the sampling areas is presented below (Table 1 and Figure 1).

### 2.2. Sample Preparation

The European hares (*Lepus europaeus*) included in this study were harvested during the hunting season (1 October to 31 December). The carcasses were processed as soon as possible immediately after hunting. Individuals selected for sampling were chosen randomly. Each specimen was assigned a unique identification number, ensuring that all data collected during sampling could be unequivocally linked to the respective individual. Sex was recorded in the field, after which one eyeball was removed for age determination and fixed in 4% formalin for 48 h [28]. Following the opening of the body cavity, the liver, kidneys, and—in the case of females—the reproductive organs were removed. From each individual, one kidney and one liver lobe were stored frozen, labelled with the corresponding individual identification number, until further analyses were conducted.

Processing and measurements of the eye lenses and reproductive organs were carried out in the laboratory of the Institute of Wildlife Management and Wildlife Biology, University of Sopron. The eye lenses were excised by an incision at the junction of the sclera and the cornea. They were then dried to constant mass in a drying oven (Memmert Model 100–800) at 105 °C for 210 min. The dry mass was subsequently determined using an analytical balance (Ohaus Explorer).

Based on the dry mass of the eye lenses, hares were classified into eleven age groups according to the methodological criteria of Suchentrunk et al. [28] (Table 2).

The methodology developed by Suchentrunk et al. [28], as described above, allows for a considerably more refined discrimination among age classes compared to the previously applied two-class system (ad.: eye lens mass < 280 mg; juv.: eye lens mass > 280 mg), as proposed by Kovács & Heltay [29].

In the collected females, the uterine horns of the reproductive tracts were longitudinally opened on the side opposite to the margo mesometricus, after which the placental scars were counted under moist conditions and recorded separately for each uterine horn. In males, testicular weights were determined, as these represent the most reliable indicators of sexually active status [25,30,31].

For comparison with the food-safety maximum levels established by Commission Regulation (EU) 2023/915 [32], we selected the liver, the edible tissue with the highest heavy metal concentrations and one that is widely consumed. For the food-safety assessment, we adopted the most conservative exposure scenario, taking into account the culinary practices associated with the consumption of European hare, including both muscle meat and edible organs. Accordingly, the liver was selected as the reference tissue for the evaluation of food-safety implications, as it generally accumulates trace elements at higher concentrations than other edible tissues [33]. Therefore, the conclusions presented in this manuscript should be interpreted within this context.

### 2.3. Analytical Procedures

Determination of heavy metal concentrations in these two organs was performed at the Laboratory of the Department of Animal Hygiene and Herd Health, University of Veterinary Medicine, using inductively coupled plasma mass spectrometry (ICP-MS). A total of 163 samples (153 kidney and 153 liver samples) were analysed. Reagents of trace-element analytical grade were used for sample preparation. Hydrogen peroxide (30% *w*/*w*, AnalaR NORMAPUR, VWR Chemicals, Leuven, Belgium), nitric acid (69% *w*/*w*, Aristar, Leuven, Belgium), hydrochloric acid (37% *w*/*w*, Aristar), and ethanol (AnalaR NORMAPUR) were purchased from VWR International Ltd. (Leicestershire, UK). Deionised water (18.2 MΩ/cm) was produced using a Purite Select Fusion 160 BP water purification system (SUEZ Water Technologies & Solutions, Trevose, PA, USA). For calibration of the inductively coupled plasma mass spectrometer (ICP-MS), a multi-element standard solution (Instrument Calibration Standard 2, TruQms) was used. Internal standard elements were added to samples, calibration standards, and blanks in the form of an Internal Standard Mix (TruQms) purchased from PerkinElmer Inc. (Waltham, MA, USA). A certified reference material (1577c Bovine Liver), obtained from Merck KGaA (Darmstadt, Germany), was used for quality control. Argon gas of 4.8 purity was purchased from Messer Hungarogáz Ltd. (Budapest, Hungary). For sample digestion, 0.5 g of each sample was weighed into CEM MARS XPress Teflon vessels. Subsequently, 5 mL of hydrogen peroxide and 5 mL of nitric acid were added, and samples were digested using a CEM MARS6 microwave digestion system (CEM Corporation, Matthews, NC, USA) under the following conditions: 35 min ramp to 200 °C, 50 min holding time, 1700 W microwave power, 40-vessel capacity.

After digestion, the contents of the Teflon vessels were transferred into 50 mL polypropylene (PP) tubes (Deltalab, Rubí, Barcelona, Spain). The solutions were then diluted to 25 mL with deionised water. Prior to analysis, samples were further diluted fivefold in 12 mL PP tubes (Deltalab, Rubí, Barcelona, Spain) after the addition of 0.4 mL ethanol and 100 µL internal standard solution. The internal standard solution contained 1 µg/mL bismuth (Bi), germanium (Ge), and indium (In). Quality control samples and blanks were prepared using the same procedure. Between digestion batches, Teflon vessels were cleaned with 0.15 M hydrochloric acid solution. Elemental concentrations were determined using a PerkinElmer NEXION 2000 inductively coupled plasma mass spectrometer (PerkinElmer, Waltham, MA, USA) operating in helium kinetic energy discrimination (KED) mode, with cell gas flow rates of 4.8–5.8 mL/min depending on the element measured. Instrumental parameters were as follows: solid-state RF generator (34.5 MHz LumiCoil), RF power 1600 W; Meinhard Plus quartz nebuliser (low internal volume, C-type); plasma gas flow rate 15 L/min; auxiliary gas flow rate 1.2 L/min; and nebuliser gas flow rate 1.06 L/min.

### 2.4. Dataset and Statistical Methods

All statistical analyses were performed in RStudio 4.5.2. [34]. The complete dataset (combining kidney and liver data via Sample ID) comprised a total of 312 observations related to the bioaccumulation of 17 trace metals (Ti, V, Cr, Mn, Fe, Co, Ni, Cu, Zn, As, Se, Mo, Sb, Cd, Sn, Hg, and Pb), measured in micrograms per gram (mg/kg). Based on age distribution, 44.9% of the individuals were classified as adults, while 55.1% were juveniles. The sex ratio consisted of 57.4% females and 42.6% males.

Decimal precision of the data was not altered. To preserve natural variability, normalization, outlier removal, substitution of half-LOD values, and other imputation procedures were not applied in our first analysis. This methodological choice was made to maintain ecological heterogeneity, as such transformations may obscure or bias rare but ecologically relevant patterns, including toxicologically significant exposure subgroups or bioaccumulation “hotspots”, as well as extreme values. However, we performed a sensitivity analysis with half-LOD values as input values for the liver-kidney data.

The dataset originates from five sampling areas: Biharkeresztes (39 individuals), Forráskút (64 individuals), Jászberény (36 individuals), Lajta (121 individuals), and Rábapordány (52 individuals). A total of 1179 cells in the dataset contained values below the limit of detection (LoD), indicated by the “<” symbol. In these cases, the symbol was removed, and the upper bound value was retained, as interval-censored data cannot be directly incorporated into statistical models. Additional variables included number (unique identifier), sample code (unique identifier combined with site code), date (sampling date, from 2023 onwards), sampling location (collection site), age (age class, available for 306 correlated samples), sex (biological sex), placental scar count (recorded for 177 samples), and organ type (liver/kidney). Organ type represents a repeated-measures factor, as each individual contributed two measurements (liver and kidney).

Descriptive statistics have already been partly reported. Inferential statistics were carried out in the following to investigate the effect of age group, sex, and organ type on the bioaccumulation of the metals separately. Separate log-linear regression models were fitted to the analytical dataset for each metal. Age class (ad. vs. juv.) was included as a fixed-effect predictor, followed by the inclusion of sex (in a separate model) and organ type (in a separate model).

To account for heteroscedasticity, robust log-linear regression models with HC3 heteroscedasticity-consistent standard errors [35] were applied to ensure reliable statistical inference, with the regression coefficients being exponentiated. The Holm–Bonferroni correction was used to control for bias arising from multiple testing across metals. Differences in the bioaccumulation of trace metal concentrations between the two soft tissues (liver and kidney), using separate GAMMs [36] again, with Holm-Bonferroni correction of *p*-values were employed as sensitivity analysis.

After every analysis, PCA (Principal Component Analysis) was carried out for visualization purposes. In addition, the correlations between the following metals, Mn & Zn, Zn & Cd, and Mn & Cu, were assessed in both organs separately using Spearman’s rank correlation analysis. K-means clustering for the five sampling sites was performed on standardized (z-transformed) values. Furthermore, K-means cluster analysis based on trace metal concentrations in relation to placental scar counts in adult female European hares was performed. Finally, we examined if trace metal profiles of adult female individuals potentially influence placental scar counts using PERMANOVA [37] with 999 permutations, using the Euclidean metric, which allows for simultaneous analysis of all measured elements. The latter was performed because the number of placental scars may act as a potential covariate, as it can be influenced by elemental bioaccumulation.

Beside inferential statistics, the Shapiro–Wilk test was applied to assess the normality of heavy metal concentration distributions. This was followed by the calculation of standard descriptive statistics as stratified summaries by age, sex, organ type, and region. Homoscedasticity was evaluated using the Breusch–Pagan test. Multicollinearity was subsequently assessed using the variance inflation factor (VIF), applying a conservative threshold of 5.

All computations were performed using the R packages *sandwich* [38,39] and *lmtest* [38]. To stabilize the skewness of and variance in the data, a natural logarithmic transformation was applied.

Following this, K-means clustering was applied to the 17 trace metals to evaluate whether the five sampling sites exhibited distinct elemental profiles based on heavy metal concentrations in soft tissues of European hares. PCA was also conducted on the dataset. Prior to analysis, all variables were standardized using z-transformation. The multivariate response consisted of all measured trace metal concentrations (Ti, V, Cr, Mn, Fe, Co, Ni, Cu, Zn, As, Se, Mo, Sb, Cd, Sn, Hg, and Pb).

Subsequently, all metal variables were z-transformed within the adult subset. The dataset was then restricted to adult individuals (*n* = 73) for the evaluation of associations between heavy metal concentrations and placental scar counts. The variables were standardised using z-transformation. The PERMANOVA model was specified as Heavy Metals ~ Age Group + Placental Scar + Age Group × Placental Scar, where the latter term represents the interaction between age group and placental scar count. For this analysis, a subset restricted to adult female individuals was evaluated. Due to the large number of dependent variables (i.e., the metals), PERMANOVA (permutational multivariate analysis of variance) was applied to assess whether the multivariate trace metal profile of adult individuals explains the variability in age group-dependent placental scar counts (Table 3). Subsequently, PCA was conducted to visualize the multivariate trace metal structure in relation to variables associated with reproductive history. Finally, we compared the different regions in terms of bioaccumulation data using the Welch test. The Welch tests were used to identify if metal differs between which specific locations. Table 3 illustrates the number of placental scars in the adult female sub-sample.

## 3. Results

First, we tested the hypothesis that the concentrations of the 17 trace metals differ according to age group. As liver tissue is metabolically active, it may accumulate higher or different amounts of trace elements than kidney tissue; therefore, adjusting for organ type helps to eliminate a major source of physiological variation that could confound the effect of age.

The Breusch–Pagan test indicated heteroscedasticity (*p* < 0.05) for several pairwise combinations of metal concentration variables, indicating that residual variance is not constant across the range of explanatory variables. VIF for most metal variables was low (VIF < 5), whereas slightly elevated multicollinearity was detected for chromium (Cr) (VIF ≈ 5.8).

In addition, a key objective was to compare the bioindicator heavy metal profiles across the sampling regions. Within the studied dataset, significant differences between age classes were detected for Ti, Fe, Cd, and Hg concentrations. In all cases, higher concentrations were observed in the adult age class. The lower metal concentrations in juveniles are reflected by negative β coefficients (Table 4).

**Figure 2:**
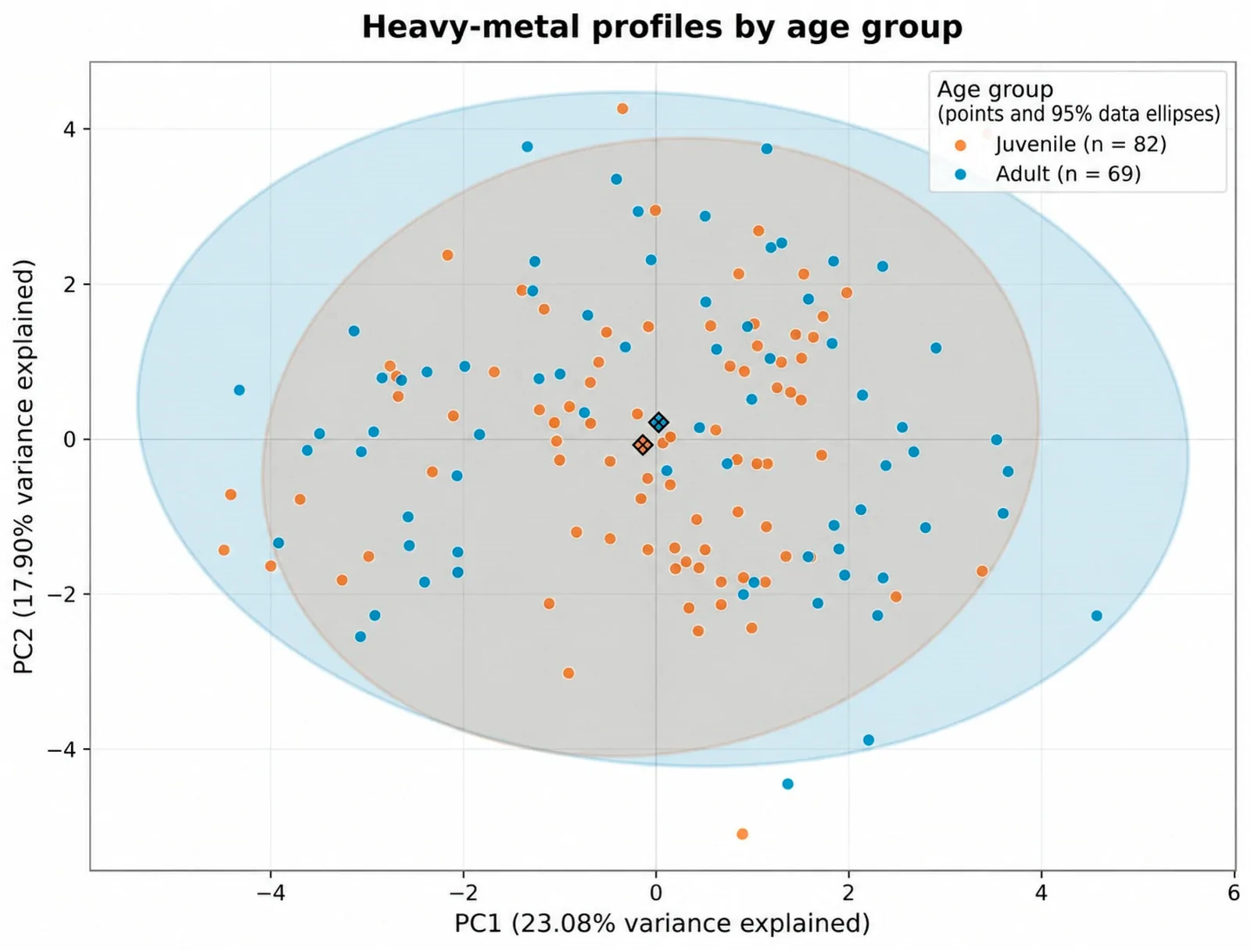
PCA visualization of Heavy Metals by Age class (adult and juvenile) based on K-means cluster analysis with pre-defined 2 clusters (adult and juvenile) of European hares. NB: The PCA provides an exploratory representation of the heavy metal profiles. The overlap between the age-group ellipses (younger and older adults) indicates limited visual separation along PC1 and PC2 but should not be interpreted as formal evidence that the groups are statistically equivalent. 95% confidence ellipse is depicted.

PCA indicated only partial multivariate separation between the two age classes based on measured trace metal concentrations. The first principal component (PC1) explained 17.04% of the total variance, while the second principal component (PC2) accounted for 13.59%, meaning that the first two dimensions together explained approximately 30.63% of the total variability. The PCA plot showed substantial overlap between the covariance ellipses of the juvenile and adult groups, indicating that the overall elemental composition patterns are largely similar between the two age classes. In other words, no robust increase in trace metal accumulation with age could be demonstrated based on the general multivariate structure (Figure 2).

**Figure 3:**
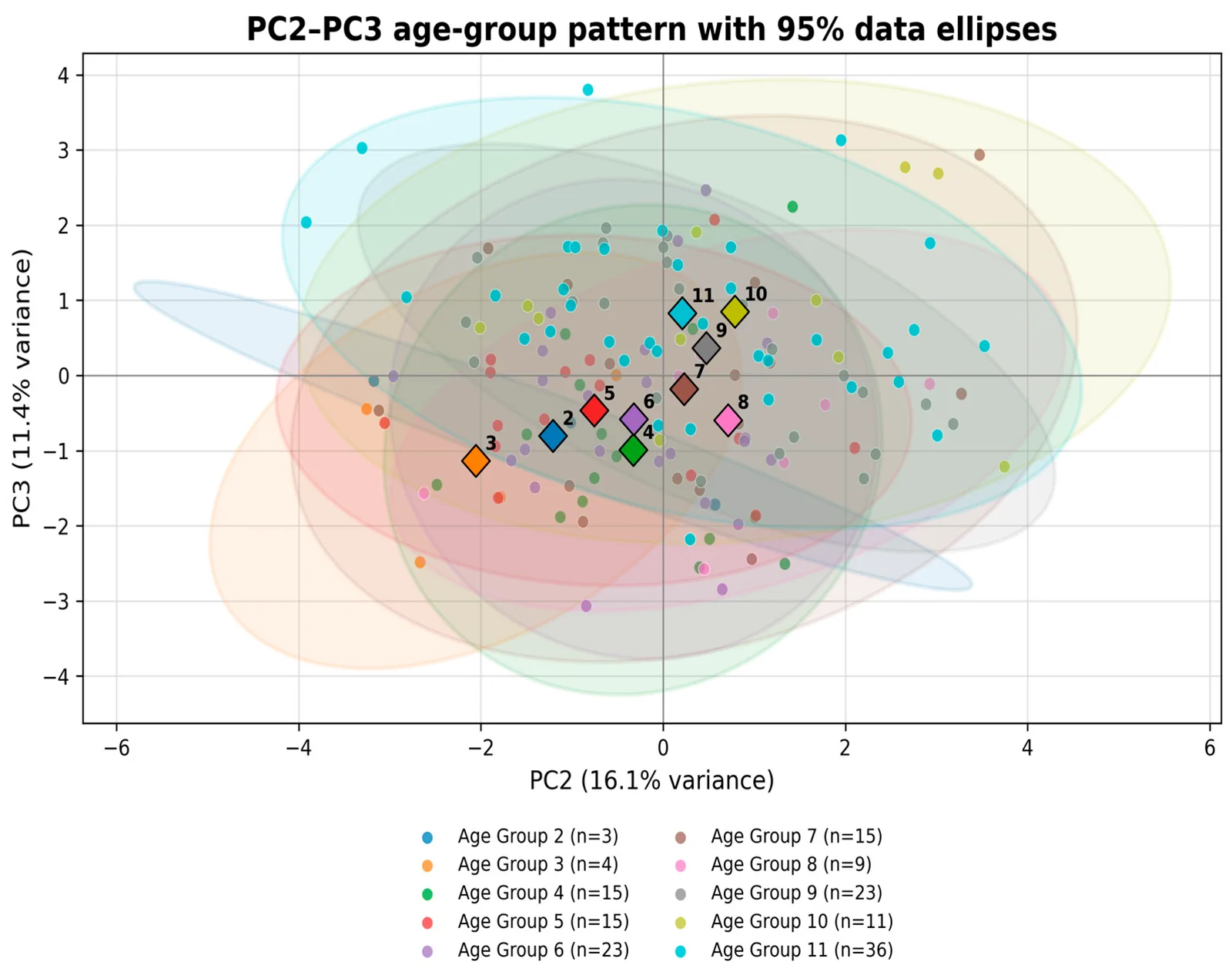
PCA of heavy metal concentrations by age group (2–11 years) in European hares, based on a k-means cluster analysis with 10 predefined clusters.

A certain degree of separation between the heavy metal profiles of the two age classes was observed, particularly along the vertical axis (PC2), indicating greater dispersion and variability in trace element profiles among older individuals. This pattern suggests that adults are more likely to exhibit elevated contaminant burdens, as more pronounced outlying metal concentrations were detected in this age group. As more detailed age data were available, the heavy metal profiles were also evaluated across ten age classes (Figure 3). The resulting pattern was consistent with the findings obtained from the comparison of the two age groups: older age classes generally occupied higher positions along the PC2 axis, although the group centroids remained relatively close to one another. This pattern was further supported by a PERMANOVA corroborated with a PERMDIST sensitivity analysis. Age group was the independent variable, with the heavy metals as the dependent variables. A one-way, Euclidean-distance-based permutational multivariate analysis of variance (PERMANOVA) [37] with 9999 permutations indicated that the kidney heavy metal concentration profile (i.e., the 17 heavy metals) differed significantly among the 10 age groups, pseudo-F(9, 144) = 1.91, *p* < 0.001, R^2^ = 0.106. Age group accounted for approximately 10.6% of the total multivariate variation in the log-transformed and standardized metal concentrations. In addition, a permutational analysis of multivariate dispersions (PERMDISP) [37] also showed a statistically significant difference in within-group multivariate dispersion, F(9, 144) = 2.64, *p* = 0.038. Thus, the age groups differed not only in the locations of their multivariate centroids but also in the degree of within-group heterogeneity.

In particular, the two youngest age groups contained only three and four animals, respectively. Their small sample sizes make their multivariate centroids and dispersion estimates relatively unstable. The lower apparent dispersion of these groups may partly be a small-sample phenomenon. Accordingly, age groups do not have different mean heavy metal profiles. Because PERMDISP was significant, pairwise PERMANOVA comparisons should not be interpreted without also examining group-specific dispersions and sample sizes. Only one statistically significant difference was detected only between age classes 4 and 11 after Holm-Bonferroni correction, which should be interpreted cautiously due to differing sample sizes and potential Type I error. Owing to the small sample sizes within the individual age classes, the variability among individuals exceeded the differences in mean heavy metal concentrations between age classes, preventing a clear and consistent separation of the groups. This observation further suggests that the exposure pattern was relatively homogeneous across the studied population, although age appears to contribute to the observed variation to some extent.

This relatively tiny cumulative proportion in the PCA analysis means that the multi-metal covariance structure was not driven by a single large gradient, but rather that variability was spread over several dimensions (Figure 2 and Figure 3). Hence, the 2D PCA map represents only a subset of the whole multivariate structure. The large overlap between the confidence ellipses for younger and older animals shows limited differentiation of the total heavy metal profiles by age along the first two major components. Older animals showed a slightly wider distribution and numerous more extreme values, especially in the direction of negative PC1 and positive PC2 values, indicating a greater interindividual variation in multi-metal accumulation. Nevertheless, the overlap between the groups was pronounced. Importantly, PCA does not show any evidence for two well separated age-related clusters because of the visible overlap. Since about 69% of the overall variance is outside PC1 and PC2. The interpretation is that the lack of strong visual distinction should not be taken as evidence that age is not associated with certain metals or with components not shown in the map.

The second hypothesis addressed whether differences exist between sexes in the concentrations of the 17 investigated trace elements. The analysis showed that no statistically significant differences could be detected between male and female European hares for any of the examined metals (Table 5).

When examining the natural logarithm–transformed concentrations of the investigated trace metals by organ type, it was found that, with the exception of Co and Hg, statistically significant differences in concentration between liver and kidney were detected for all elements (Table 6).

We ran a sensitivity analysis using not the raw values but half of them (LOD/2). We opted for separate GAMMs (Generalized Additive Mixed Models) [36] as sensitivity analysis. Individual, Sex, Age Group, and Sampling Location were entered as random effects, as they can potentially affect the level of bioaccumulation in the individuals. Holm-Bonferroni-corrected significance values were reported (Table 7).

**Figure 4:**
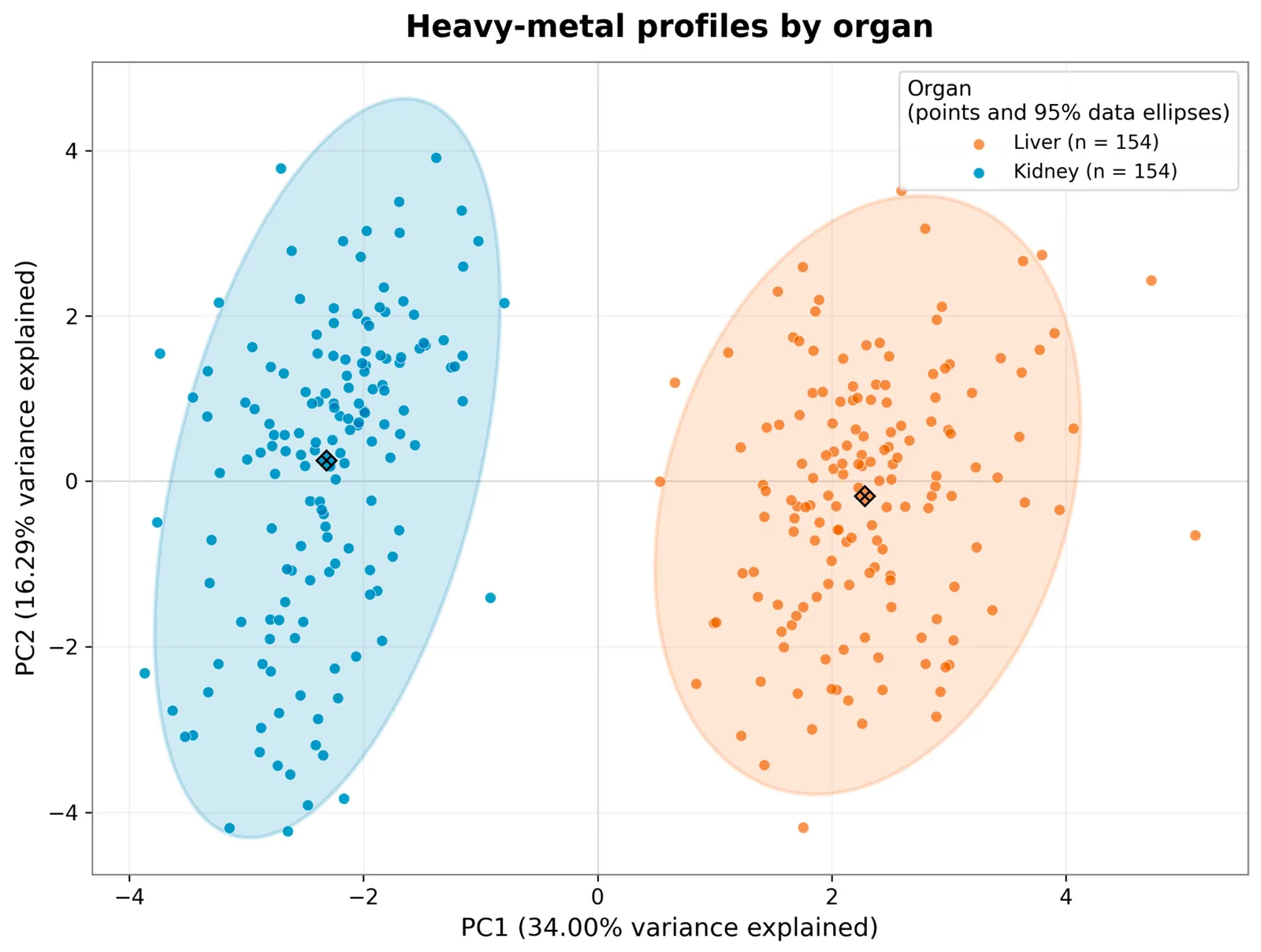
PCA visualization of the distribution of data points-based k-means cluster analysis (2 clusters: kidney and liver) based on trace metal concentrations in the soft tissues of European hares (kidney and liver). 95% confidence ellipse is depicted.

PCA analysis performed on liver and kidney samples showed that the first two principal components together explained approximately 41.79% of the total multivariate variability (PC1: 27.84%; PC2: 13.95%), indicating that the PCA space is suitable for visualizing trace metal concentrations in soft tissues. The PCA plot revealed a clear horizontal separation between the two examined organs along PC1. Kidney samples clustered predominantly in the negative range of PC1, whereas liver samples were mainly located at positive PC1 values. This pattern strongly suggests that the elemental composition differs systematically between the two organs. In other words, multivariate trace metal profiles are well separated by organ type. Liver samples exhibited greater dispersion along the horizontal axis, indicating higher variability in trace metal accumulation patterns within this tissue type. Importantly, the PCA results indicate that the overall multivariate elemental composition differs between the two organs (Figure 4).

Given that associations between Mn and Zn, as well as between Zn and Cd concentrations, are well documented in physiological contexts, we further examined the relationships between the measured values in our dataset. In liver tissue, a statistically significant positive Spearman correlation was found between manganese (Mn) and zinc (Zn) concentrations (ρ = 0.458, *p* < 0.001). A significant positive correlation was also observed between manganese (Mn) and copper (Cu) concentrations in the liver (ρ = 0.251, *p* = 0.001), although the strength of this association ranged from weak to moderate. The same Spearman correlations were evaluated in kidney tissue. In the kidney, a significant positive correlation was identified between manganese (Mn) and zinc (Zn) concentrations (ρ = 0.352, *p* < 0.001), with a moderate effect size. Similarly, a statistically significant positive Spearman correlation was found between manganese (Mn) and copper (Cu) concentrations (ρ = 0.503, *p* < 0.001), with the association ranging from moderate to strong. Spearman correlation analysis further revealed a weak but statistically significant positive association between zinc (Zn) and cadmium (Cd) concentrations in the liver (ρ(157) = 0.167, *p* = 0.035). The same analysis performed for kidney samples showed a moderate positive correlation between Zn and Cd concentrations (ρ(155) = 0.272, *p* < 0.001).

We examined whether statistically significant differences in trace metal concentrations of European hare samples could be detected among the sampling sites. Tin (Sn) was excluded from the analysis, as it showed no variability: all measured values were <0.18 across all samples. This indicates identical descriptive statistics across sites (with mean values approximately 0.225), and therefore no statistical analysis was performed due to the absence of variability. No statistically significant differences among sampling sites were detected for Cr, Fe, Cu, and Mo. The lowest, yet statistically significant, degree of spatial variation was observed for Ti and Ni. These elements were followed by Sb, Zn, and Mn in terms of increasing between-site differences. Higher levels of spatial heterogeneity were observed for Cd, V, and As, while the greatest variability among sampling locations was found for Co, Hg, Pb, and Se. For Se, statistically significant differences were detected between nearly all sampling sites (Table 8).

**Figure 5:**
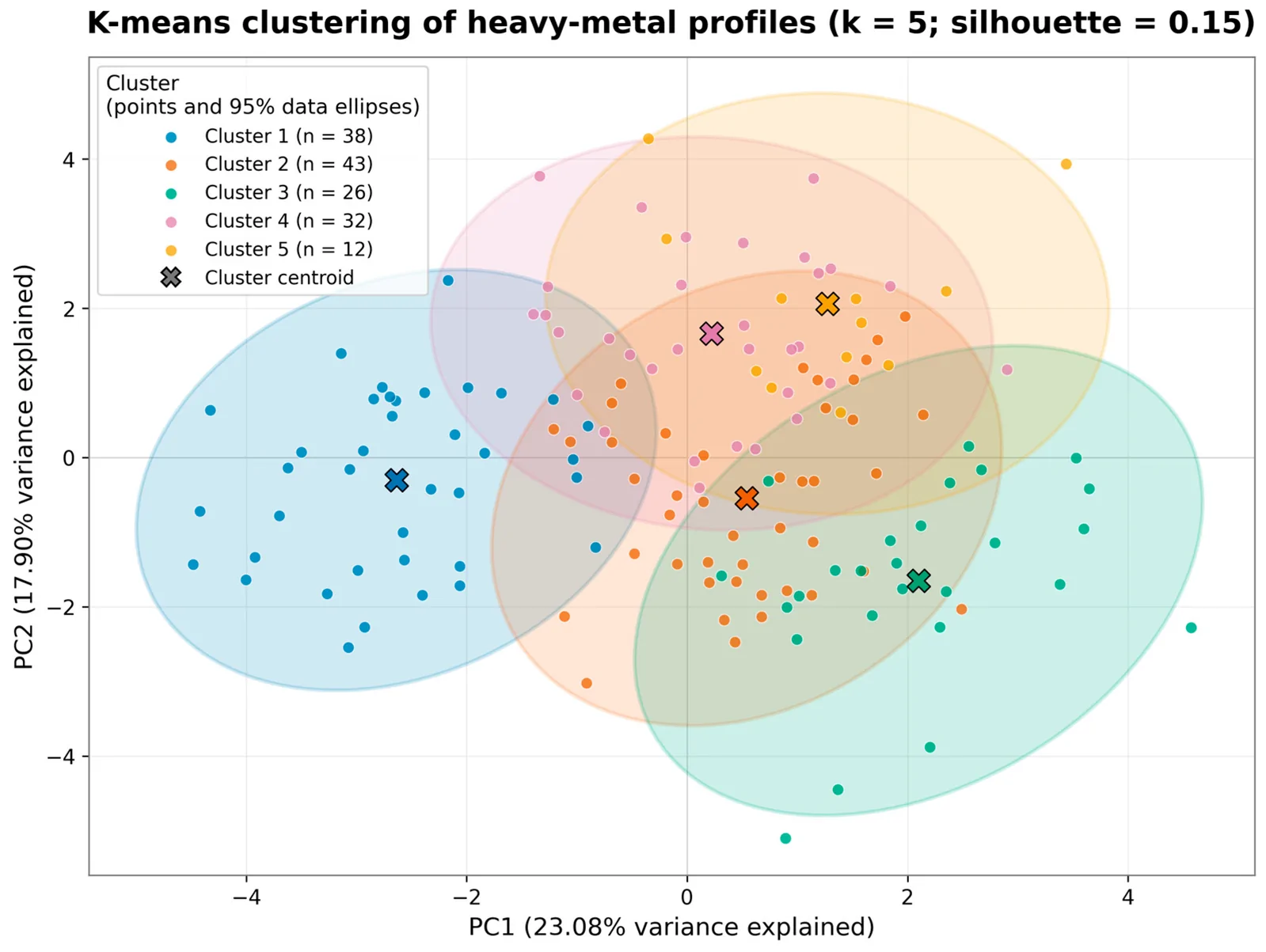
PCA-based visualization of k-means cluster analysis (5 clusters) based on trace metal concentrations. The five clusters represent sampling locations. 95% confidence ellipses are depicted.

K-means clustering for the five sampling sites was performed on standardized (z-transformed) values. The analysis included 158 European hare individuals and the 17 trace metal variables detected in their tissues, which were simultaneously included in the clustering procedure. In the PCA representation, the first principal component explained 20.57% of the variance, while the second accounted for 19.51%. The five-cluster solution yielded a total within-cluster inertia of 1703.26, indicating a moderate level of within-cluster dispersion. This suggests that trace metal profiles are not fully homogeneous within clusters derived from the five sampling areas; however, some degree of spatial differentiation is still apparent. This pattern is also reflected by the silhouette coefficient (0.102), which measures cluster separation and compactness. This relatively low value indicates weak separation between clusters, with many samples located near cluster boundaries. Consequently, trace metal profiles tend to form gradual transitions rather than clearly distinct groups. Overall, elemental composition patterns indicate gradual rather than discrete spatial variation visually, meaning that trace metal profiles of the sampling sites do not show strong separation based on concentrations measured in hare tissues (Figure 5). Importantly, The PCA did not reveal pronounced visual separation between the groups along the first two components.

**Figure 6:**
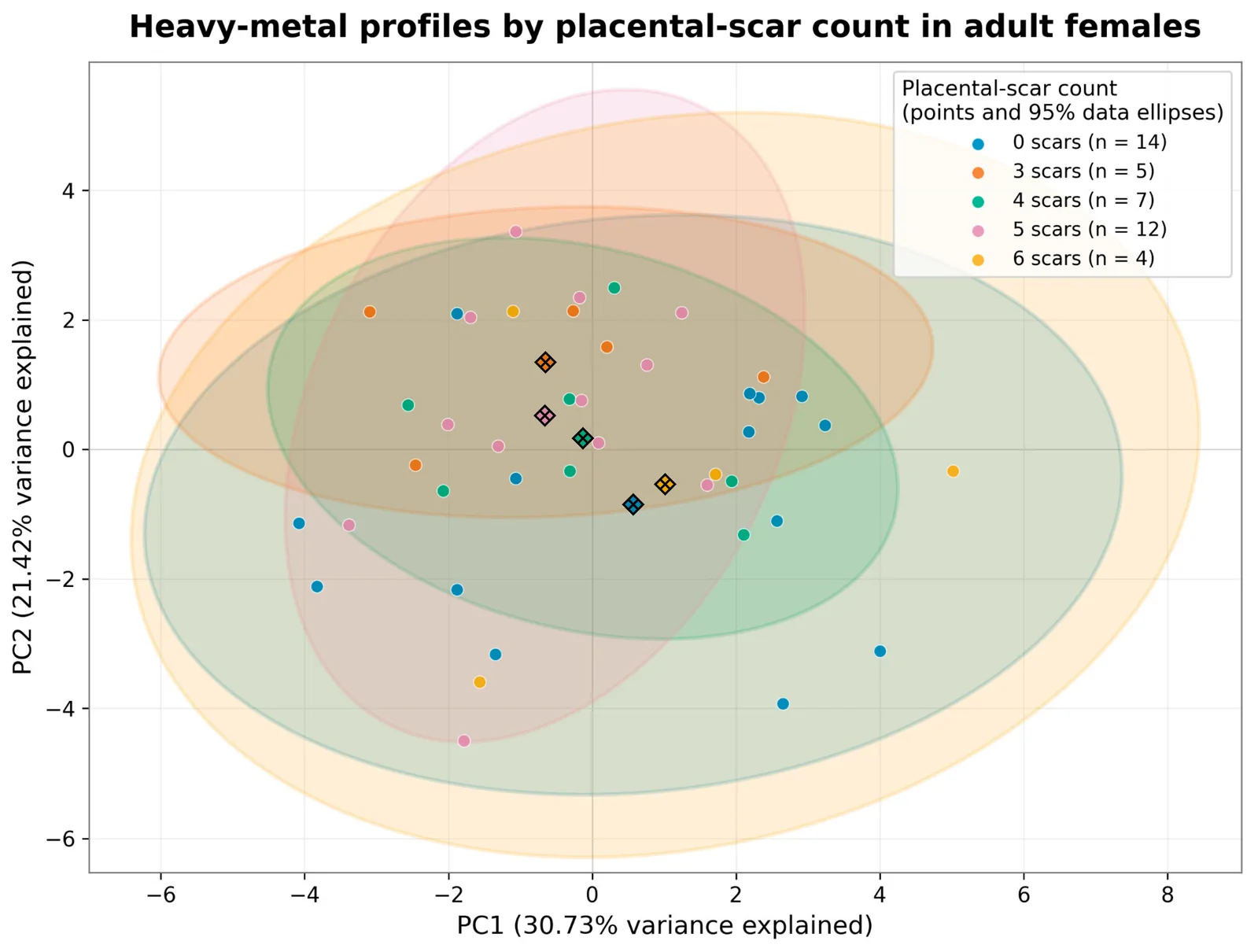
PCA-based visualization of k-means cluster analysis of placental scars based on trace metal concentrations in relation to placental scar counts in adult female European hares. 95% confidence ellipse is depicted.

We next examined, employing PERMANOVA, whether trace metal concentrations have an effect on the number of placental scars recorded across age classes (9–11). The results indicate that, even when accounting for interaction effects, the trace metal profiles of adult female individuals do not influence placental scar counts. The effect of age group approached did not reach the significance threshold of α = 0.05, (F(2, 33) = 1.59; *p* = 0.060; R^2^ = 0.063), using PERMANOVA again. The effect of placental scar count was not statistically significant (F(4, 33) = 0.97; *p* = 0.519; R^2^ = 0.077, PERMANOVA). Likewise, the interaction between age group and placental scar count was not significant, (F(6, 33) = 1.25; *p* = 0.182; R^2^ = 0.148, PERMANOVA) (Figure 6).

## 4. Discussion

Based on the variance inflation factor (VIF ≈ 5.8) for the 17 trace metals analysed above, no multicollinearity was detected, indicating that accumulation processes for individual metals can be interpreted independently. At the same time, we acknowledge previously reported associations in the literature [40], particularly between Zn and Cd, as well as Mn and Cu.

Mercury (Hg) is a neurotoxic and nephrotoxic heavy metal [41] that has no known physiological function in mammals [26]. Long-term mercury exposure has been shown to disrupt the antioxidant defence system in brown hares by altering glutathione metabolism and antioxidant enzyme activities, suggesting the induction of oxidative stress as a potential mechanism of mercury toxicity [42]. The last available Hungarian data on Hg in European hare date back to 1976, when mean concentrations of 1.7 mg/kg (*n* = 9) in liver and 4.9 mg/kg (*n* = 9) in kidney were reported in hares collected near crops treated with mercury-based fungicides [21], substantially exceeding current regulatory limits. The use of mercury-containing pesticides has since been prohibited; therefore, such extreme values are no longer reported in recent studies. In Serbia, kidney concentrations ranging from 0.029 to 0.051 mg/kg and liver concentrations from 0.013 to 0.028 mg/kg have been reported depending on age class. A significant difference in hepatic Hg concentrations was observed between 24–36-month-old and 12-month-old individuals (*p* < 0.05), while no significant differences were detected among other age groups [43]. In contrast, Slovak studies reported no significant relationship between age and Hg concentrations in liver and kidney tissues [44]. In Turkey, mean Hg concentrations of 0.10 ± 0.12 mg/kg in kidney samples and 0.06 ± 0.03 mg/kg in liver samples (*n* = 15) were reported [33], values which are considerably higher than those observed in the present study (kidney: 0.0248 ± 0.0233 mg/kg; liver: 0.02 ± 0.0092 mg/kg). Massányi et al. [44] detected significantly higher Hg concentrations in winter-collected hare samples, an observation of particular relevance to the hunting season in Hungary.

Lead (Pb) is a toxic heavy metal affecting the hematopoietic system, kidneys, and nervous system. In a rabbit model, lead exposure caused macrocytic hyperchromic anaemia, increased AST activity, and reduced pancreatic enzyme activities, demonstrating toxic effects on the haematopoietic system, liver, and pancreas [45,46]. In Croatia, elevated Pb exposure has been shown to negatively affect oxidative status in European hares, potentially inducing toxic effects on the brain and muscle tissue, and may represent one of the contributing factors to population decline [47]. Gál [20] reported mean Pb concentrations of 0.39 mg/kg in liver samples from hares in the Kisalföld region. In Turkish samples, Pb concentrations of 1.23 ± 1.22 mg/kg in kidney and 2.19 ± 2.87 mg/kg in liver were reported [33]. In Slovak populations, seasonal comparisons showed that median Pb concentrations in liver and kidney were highest during winter and significantly exceeded values recorded in spring, summer, and autumn (*p* < 0.001) [44]. No significant relationship between age and Pb concentrations was observed, consistent with the findings of the present study. In contrast, sex-based comparisons revealed significantly higher hepatic Pb concentrations in males (0.216 mg/kg) than in females (0.127 mg/kg) (*p* < 0.05) [44] a pattern not supported by our results.

Cadmium (Cd) is a highly cumulative heavy metal that damages the kidney, liver, and reproductive and skeletal systems. Its accumulation has been associated with histological alterations in the ovaries and uterus, indicating its adverse effects on female reproductive tissues [48]. In a rabbit model, cadmium exposure induced mild renal tubular damage, increased ALT activity, and reduced pancreatic enzyme activities, indicating adverse effects on renal, hepatic, and pancreatic function [46]. Due to its long biological half-life, it primarily accumulates in the liver and kidneys [49,50]. Gál [20] reported Cd concentrations of 0.158 mg/kg in juvenile hares and 0.2 mg/kg in the liver of adult European hares. In Serbian hare populations, kidney Cd concentrations were significantly higher than liver concentrations across all age groups, and increasing concentrations were observed in both organs with advancing age [43]. The authors demonstrated a strong relationship between renal Cd levels and age, supporting the bioaccumulative nature of cadmium. Slovak studies also reported significantly higher Cd concentrations in the liver and kidneys of adults compared to juveniles, although no statistically significant differences were observed between sexes [44]. In the present study, significant differences were observed with respect to sex, age, and both examined soft tissues. Based on these findings, the European hare can be considered a suitable bioindicator species for monitoring environmental Cd exposure, as tissue concentrations reliably reflect long-term environmental exposure [43].

Zinc (Zn) is an essential trace element involved in numerous enzymatic processes, protein synthesis, immune regulation, and antioxidant defence mechanisms [51]. In European hare studies, Zn predominantly accumulates in the liver and kidneys. Polish investigations reported mean concentrations of 31.25 mg/kg in liver and 26.49 mg/kg in kidney, which are in good agreement with the values obtained in the present study (kidney: 27.974 mg/kg; liver: 38.8337 mg/kg). The relationship between Zn and Cd is physiologically antagonistic, as they share partially overlapping absorption and binding pathways in the organism [52]. However, some studies have reported positive correlations between tissue Zn and Cd concentrations, likely due to the induction of metallothionein proteins involved in their co-sequestration [40]. Our results support these findings, as a weak positive correlation in the liver and a moderate positive correlation in the kidney were observed between Zn and Cd concentrations.

Manganese (Mn) is an essential microelement involved in numerous enzymatic systems, carbohydrate and lipid metabolism, bone formation, and antioxidant defence. In mammals, Mn is primarily found in the liver, kidneys, and bone tissue, with its concentration tightly regulated by homeostatic mechanisms [53,54]. As a result, Mn typically exhibits lower variability than many toxic heavy metals; however, environmental conditions, diet composition, and inter-element interactions may influence its accumulation. In Polish studies on European hares, mean Mn concentrations of 2.0 mg/kg in kidney and 2.51 mg/kg in liver (wet weight basis) were reported. Significant positive correlations between Mn and Zn (rs = 0.72), as well as Mn and Cu (rs = 0.79), were detected in liver tissue, highlighting metabolic interactions among essential trace elements [40]. These associations were also confirmed in both liver and kidney samples in the present study. In contrast, higher Mn concentrations were reported in Turkish hare populations (6.00 mg/kg in kidney and 4.80 mg/kg in liver; [33] where accumulation was highest in kidneys, differing from several European studies reporting higher hepatic concentrations. A more recent Serbian study analysing 157 European hares reported mean Mn concentrations of 1.75 mg/kg in kidney and 2.36 mg/kg in liver, with no significant age-related differences [54]. In the present study, mean Mn concentrations were 2.6458 ± 0.7188 mg/kg in liver and 2.1064 ± 0.3366 mg/kg in kidney, consistent with Polish and Serbian results but contrasting with Turkish findings.

Iron (Fe) is one of the most important essential trace elements in the body, playing a fundamental role in oxygen transport, cellular respiration, and numerous enzymatic processes. In mammals, iron is predominantly stored in the liver [53]. A Serbian study analysing 157 European hare liver and kidney samples reported mean Fe concentrations of 103.3 ± 42.1 mg/kg in kidney and 138.5 ± 52.7 mg/kg in liver, with no significant age-related differences. Mysłek and Kalisińska [40] reported average Fe concentrations of 119 mg/kg in kidney and 198 mg/kg in liver, while juvenile liver concentrations reached up to 236 mg/kg. In the present study, mean concentrations were 92.7541 ± 37.7116 mg/kg in kidney and 162.4372 ± 108.1145 mg/kg in liver. The maximum observed liver concentration reached 912.4517 mg/kg.

Nickel (Ni) is a natural component of soil; however, its concentration may increase significantly in the environment due to industrial emissions [55]. In mammals, nickel primarily accumulates in the liver and kidneys, and at elevated levels it may exert toxic and potentially carcinogenic effects [56]. Wajdzik et al. [57] demonstrated a tissue-specific distribution of nickel in the European hare. The highest concentrations were observed in brain tissue (5.9 mg/kg), whereas the lowest values were detected in bone tissue (0.32 mg/kg). In the present study, mean concentrations of 0.1038 ± 0.0738 mg/kg in kidney and 0.2458 ± 0.1271 mg/kg in liver were measured. As part of the general health assessment of the studied hares, macroscopic pathological lesions were also recorded during necropsy. No organ lesions suggestive of carcinoma were detected in any of the individuals examined and subjected to heavy metal analysis [25].

Copper (Cu) is an essential trace element and a component of numerous oxidoreductase enzymes, playing a key role in iron metabolism, hematopoiesis, and antioxidant defence systems [51,53]. In the European hare, copper predominantly accumulates in the liver, which serves as its primary storage and metabolic organ. In a Serbian population study, Petrović et al. [54] reported mean Cu concentrations of 3.32 ± 0.62 mg/kg in kidney and 4.16 ± 1.40 mg/kg in liver, with significantly higher values in hepatic tissue. The authors did not detect significant age-related differences in copper accumulation. In the present study, mean concentrations of 4.0712 ± 0.45 mg/kg in kidney and 5.8504 ± 4.9016 mg/kg in liver were measured, with a maximum hepatic concentration of 63.5541 mg/kg. According to Hungarian regulations, the maximum permissible level in game meat and its products is 5 mg/kg; this threshold was exceeded by mean liver concentrations, although liver is also considered a culinary delicacy among hunters.

Arsenic (As) is a non-essential element that is toxic even at low concentrations. Long-term exposure is associated with carcinogenic, mutagenic, and cytotoxic effects. Bukovjan et al. [58] quantified arsenic concentrations in various tissues of 105 European hares. Mean concentrations of 0.019 mg/kg in liver and 0.028 mg/kg in kidney indicated higher accumulation in renal tissue. The highest concentrations were detected in brain tissue (0.389 mg/kg), fur (0.724 mg/kg), and faeces (0.375 mg/kg). The authors did not observe significant sex-related differences in arsenic accumulation, consistent with our findings. In Hungarian samples, mean concentrations of 0.0131 ± 0.0077 mg/kg in kidney and 0.0062 ± 0.0029 mg/kg in liver were measured, which are lower than those reported in Czech studies, but confirm higher accumulation in the kidney. Significant differences between soft tissues were also supported by our results, whereas no statistically significant differences were observed between age groups.

Selenium (Se) is an essential trace element that, as a component of various selenoproteins, plays a key role in antioxidant defence, immune regulation, and thyroid hormone metabolism [51,53]. In the European hare, selenium primarily accumulates in the liver and kidneys. In Polish populations, Mysłek and Kalisińska [40] reported mean concentrations of 0.42 mg/kg in liver and 0.58 mg/kg in kidney, indicating higher accumulation in renal tissue. The ecological and toxicological relevance of selenium is enhanced by its interactions with toxic elements, particularly mercury, whose harmful effects may be partially mitigated through the formation of stable selenium–mercury complexes. Consequently, selenium status may play an important role in moderating the effects of environmental heavy metal exposure. In the present study, mean concentrations of 1.1804 ± 0.3597 mg/kg in kidney and 0.4619 ± 0.4053 mg/kg in liver were measured, further confirming higher accumulation in the kidney.

Cobalt (Co), as a component of vitamin B12, is essential for proper hematopoiesis and energy metabolism [51]. In herbivorous mammals, cobalt availability is primarily determined by the mineral composition of soil and vegetation [53]. In wildlife species, cobalt generally occurs at low concentrations; however, tissue levels may serve as a reliable indicator of environmental trace element availability [59]. To date, no published data are available regarding cobalt in the European hare. In the present study, mean cobalt concentrations of 0.045 ± 0.0243 mg/kg in kidney and 0.0407 ± 0.0215 mg/kg in liver were measured, indicating similar tissue distribution between the two organs.

Molybdenum (Mo) is an essential trace element acting as a cofactor in several oxidoreductase enzymes involved in purine and sulfur-containing compound metabolism [51]. The molybdenum balance in the organism is primarily regulated by the liver and kidneys; therefore, these organs provide important information for assessing environmental exposure and element metabolism. No previous data are available on molybdenum in the European hare. In the present study, mean concentrations of 0.498 ± 0.1139 mg/kg in kidney and 1.0601 ± 0.2117 mg/kg in liver were measured, indicating higher accumulation in hepatic tissue.

Chromium (Cr) is a trace element occurring in the environment from both natural and anthropogenic sources. Trivalent chromium (Cr^3+^) may play a role in carbohydrate and lipid metabolism in mammals, whereas hexavalent chromium (Cr^6+^) is known to be highly toxic and carcinogenic [55,60]. Chromium is mainly introduced into the organism via food and accumulates primarily in the liver and kidneys, and to a lesser extent in muscle tissue. Literature on chromium in European hare is scarce at both national and international levels. In Turkish hare samples, mean concentrations of 1.02 ± 0.90 mg/kg in kidney and 1.00 ± 0.78 mg/kg in liver were reported [33], which differ substantially from the values obtained in the present study, both in kidney (0.0294 ± 0.0093 mg/kg) and liver (0.1455 ± 0.1159 mg/kg). These differences may indicate substantial variation in environmental exposure and highlight the importance of systematic bioindication monitoring.

Titanium (Ti) is one of the most abundant elements in the Earth’s crust; however, its biological role in mammals remains unclear. It enters the organism mainly via food and environmental dust, but typically accumulates only at low concentrations in tissues [55]. In wild mammals, titanium levels are primarily influenced by local soil conditions and anthropogenic environmental pressure [59]. Due to its herbivorous feeding behaviour, the European hare may serve as a suitable bioindicator of environmental titanium exposure; however, no studies have been conducted on this element in hares either in Hungary or internationally. In the present study, mean concentrations of 0.1403 ± 0.0776 mg/kg in kidney and 0.2424 ± 0.1906 mg/kg in liver were measured.

Vanadium (V) is a naturally occurring trace element in the environment; however, its concentration may increase significantly due to industrial emissions and fossil fuel combustion [55]. At elevated concentrations, it may induce oxidative stress as well as hepatic and renal toxicity in mammals [61]. Vanadium primarily accumulates in the liver, kidneys, and bone tissue; therefore, these organs are suitable for assessing environmental exposure [51]. No previous studies are available on vanadium in European hare. In the present study, mean concentrations of 0.0181 ± 0.0124 mg/kg in kidney and 0.015 ± 0.0111 mg/kg in liver were determined, providing a potential baseline for future international comparisons.

Tin (Sn) is a non-essential element for mammals and generally occurs at low concentrations in tissues [55]. No comparable literature data are available for this element in European hare. In the present study, concentrations of 0.18 mg/kg in kidney and 0.27 mg/kg in liver were measured.

Although several studies have performed between-country comparisons of heavy metal exposure [33,40,44,54,62], within-country spatial differences in contamination profiles using the European hare as a bioindicator have not been investigated. Therefore, our results cannot be directly compared with existing international datasets. Reproductive biology analyses conducted on the same dataset indicated that no changes have occurred in reproductive parameters compared to earlier literature data [25]; thus, our findings do not support an effect of heavy metal exposure on reproduction. Although climate change has resulted in an extended breeding season with potentially more favourable reproductive conditions [25], habitat quality (e.g., low crop diversity and the predominance of crops unsuitable for the European hare), combined with predation pressure, is likely to be the principal limiting factor for population performance, rather than the toxic effects of chemicals associated with intensive agricultural management.

## 5. Conclusions

Based on our results, the European hare may serve as an important bioindicator species for assessing heavy metal contamination in terrestrial habitats from multiple perspectives. The species has an extensive distribution range, occurring natively from Western Europe to the West Siberian Plain and Southwest Asia (Iran). The eastern boundary of its range reached only the Volga River at the beginning of the 19th century; however, within approximately 150 years, it expanded as far as Lake Baikal. It has also been introduced across the continent to Ireland and Scandinavia, the Far East, Australia and New Zealand, and North and South America [29,63,64]. As a widely hunted species throughout Europe, sample collection is logistically straightforward, enabling comparative assessments across broad geographic regions using standardized methodologies. Given that agricultural landscapes represent ecosystems subject to significant environmental pressure [65,66], and that this species is also relevant from a food-safety perspective [14], the investigation of heavy metal exposure in this context is of particular importance. Furthermore, the presence of substantial exposure may warrant additional complex chemical analyses of both water resources and soils. We consider it important to note that we do not have environmental monitoring data (e.g., soil, water, or vegetation measurements) on heavy metal contamination from the sampling sites. Therefore, we are unable to establish a direct causal relationship between agricultural activities and the accumulation of trace elements in European hares. Accordingly, our findings should be interpreted as an indicator of the environmental status of intensive agricultural landscapes, based on the bioindicator role of the European hare, rather than as evidence of a direct cause-and-effect relationship. Nevertheless, studies of this type can contribute to a better understanding of the bioaccumulation and biomagnification of heavy metals, as well as their ecological and toxicological effects.

The maximum permissible heavy metal concentrations in foods marketed in Hungary were previously regulated by Decree 17/1999 (VI. 16.) of the Ministry of Health (EüM) [67]. This legislation included specific provisions for game meat and game meat products, establishing maximum concentrations for As, Hg, Pb, Cd, Cu, and Zn. The currently applicable Hungarian Ministry of Rural Development Decree 49/2014 (IV. 29.) [68] establishes a maximum permissible concentration only for copper (Cu), setting the limit at 5 mg/kg, consistent with the value specified in the former Ministry of Health regulation. Based on our results, hepatic Cu concentrations exceeded this threshold in only 58.49% of the examined individuals. Considering that previous international studies have reported liver Cu concentrations to be up to 6.7 times higher than those measured in skeletal muscle in the European hare [33], it is highly likely that the meat itself complies with the applicable food-safety limits. However, from the perspective of heavy metal exposure, the consumption of visceral organs—particularly the liver—may warrant greater caution. The currently effective Decree 49/2014 (IV. 29.) of the Ministry of Rural Development (VM) [68] specifies a limit value only for Cu, maintaining the same threshold as the former EüM regulation, namely 5 mg/kg. Based on our results, the mean Cu concentration measured in liver samples exceeded this regulatory limit.

The currently applicable Commission Regulation (EU) 2023/915 [32], which establishes maximum levels for certain contaminants in foodstuffs, does not set specific limits for the aforementioned heavy metals in game meat and game meat products. Given that the European brown hare (*Lepus europaeus*) is the most widely marketed small game species for human consumption in Hungary, our findings suggest that this species may serve as an important bioindicator for assessing heavy metal contamination in agricultural habitats. Consequently, the results of the present study may provide a valuable scientific basis for the future establishment of food-safety threshold values for heavy metals in game meat.

## Figures and Tables

**Table 1:** Geographic distribution of sampling sites.

	Region	Locality	Sample Size (*n*)
**Kidney**	Little Hungarian Plain	Rábapordány	26
		Mosonszolnok	60
	**Sum.**		**86**
	Great Hungarian Plain	Jászberény	19
		Forráskút	32
		Biharkeresztes	20
	**Sum.**		**71**
**Liver**	Little Hungarian Plain	Rábapordány	26
		Mosonszolnok	61
		Vasegerszeg	4
	**Sum.**		**91**
	Great Hungarian Plain	Jászberény	17
		Forráskút	32
		Biharkeresztes	19
	**Sum.**		**68**

**Table 2:** Age classes of European hare based on dry eye lens mass [28].

Dry Eye Lens Weight (mg)	Estimated Age (Months)	Age Group Number	Age Class
25–100	1>	1	Juvenile
101–125	1–2	2	
126–150	2–3	3	
151–175	3–4	4	
176–200	4–6	5	
201–225	6–7	6	
226–250	7–9	7	
251–280	9–10	8	Adult
281–340	14–23	9	
341–360	26–35	10	
361<	38–47<	11	

**Table 3:** Number of placental scars in the adult female subset.

Age Group	Placental Scars	No. of Individuals
9	0	3
9	3	3
9	4	4
9	5	2
9	6	3
10	0	1
10	3	2
10	5	4
11	0	10
11	3	1
11	4	4
11	5	7
11	6	2

**Table 4:** Differences in log-transformed trace metal concentrations between age classes (Adult vs. Juvenile).

Metal (mg/kg)	β	SE (HC3)	t	CI Lower	CI Upper	*p*	exp(β)	*p*-Holm
Ti	−0.2730	0.0635	−4.3015	−0.3974	−0.1486	<0.001	0.7611	<0.001
V	0.0683	0.0793	0.8613	−0.0871	0.2236	0.3891	1.0707	>0.99
Cr	0.0197	0.0921	0.2142	−0.1608	0.2003	0.8304	1.0199	>0.99
Mn	0.0576	0.0283	2.0332	0.0021	0.1131	0.0420	1.0593	0.4620
Fe	−0.2495	0.0580	−4.2986	−0.3632	−0.1357	<0.001	0.7792	<0.001
Co	−0.0546	0.0752	−0.7266	−0.2019	0.0927	0.4675	0.9469	>0.99
Ni	0.0015	0.0873	0.0177	−0.1695	0.1726	0.9859	1.0015	>0.99
Cu	0.0665	0.0290	2.2949	0.0097	0.1233	0.0217	1.0688	0.2604
Zn	−0.0036	0.0297	−0.1228	−0.0618	0.0545	0.9023	0.9964	>0.99
As	0.0081	0.0686	0.1181	−0.1264	0.1426	0.9060	1.0081	>0.99
Se	−0.1737	0.1008	−1.7229	−0.3714	0.0239	0.0849	0.8405	0.8490
Mo	−0.0089	0.0502	−0.1776	−0.1074	0.0895	0.8590	0.9911	>0.99
Sb	0.0759	0.0730	1.0388	−0.0673	0.2190	0.2989	1.0788	>0.99
Cd	−1.1525	0.1588	−7.2565	−1.4638	−0.8412	<0.001	0.3158	<0.001
Hg	−0.3366	0.0741	−4.5397	−0.4819	−0.1913	<0.001	0.7142	<0.001
Pb	0.0076	0.1015	0.0750	−0.1913	0.2065	0.9402	1.0076	>0.99

Note. The two-sided *p*-values reflect Holm-Bonferroni correction applied across 16 comparisons (Sn did not show any variation, hence not reported in the table). The concentrations of individual metals were modelled on the natural logarithmic scale as a function of age. Standard errors are heteroscedasticity-consistent (HC3) and clustered at the individual level, approximating the specified random-effects structure. The coefficient β represents the unstandardized regression coefficient on the log scale.

**Table 5:** Differences in log-transformed trace metal concentrations between sexes (♀ × ♂).

Metal (mg/kg)	β	SE (HC3)	t	CI Lower er	CI Upper	*p*	exp(β)	*p*-Holm
Ti	−0.055	0.066	−0.8333	−0.1845	0.0744	0.4047	0.9465	>0.99
V	−0.2315	0.0785	−2.9499	−0.3853	−0.0777	0.0032	0.7934	0.054
Cr	−0.04	0.0942	−0.4243	−0.2245	0.1446	0.6713	0.9608	>0.99
Mn	0.0113	0.0295	0.384	−0.0465	0.0692	0.701	1.0114	>0.99
Fe	0.0591	0.0592	0.9986	−0.0569	0.1752	0.318	1.0609	>0.99
Co	−0.1491	0.0765	−1.9491	−0.299	0.0008	0.0513	0.8615	0.8718
Ni	−0.0734	0.0889	−0.8252	−0.2477	0.1009	0.4092	0.9292	>0.99
Cu	−0.0375	0.0291	−1.2877	−0.0946	0.0196	0.1978	0.9632	>0.99
Zn	−0.0001	0.0303	−0.0025	−0.0595	0.0593	0.998	0.9999	>0.99
As	−0.0697	0.0664	−1.05	−0.1999	0.0604	0.2937	0.9327	>0.99
Se	0.0906	0.1024	0.8843	−0.1102	0.2913	0.3765	1.0948	>0.99
Mo	−0.0331	0.0496	−0.6683	−0.1303	0.064	0.5039	0.9674	>0.99
Sb	−0.0535	0.0719	−0.744	−0.1944	0.0874	0.4569	0.9479	>0.99
Cd	−0.232	0.1694	−1.3694	−0.564	0.1	0.1709	0.793	>0.99
Sn	−0.0004	0.0234	−0.0162	−0.0463	0.0455	0.9871	0.9996	>0.99
Hg	0.0462	0.0763	0.6048	−0.1034	0.1957	0.5453	1.0472	>0.99

Note. The *p*-values reflect Holm-Bonferroni correction applied across 17 comparisons. The concentrations of individual metals were modelled on the natural logarithmic scale as a function of sex. Standard errors are heteroscedasticity-consistent (HC3) and clustered at the individual level, providing robustness and approximating the specified random-effects structure. Two-sided *p*-values were adjusted using Holm-Bonferroni correction across the 17 tests. The coefficient β represents the unstandardized regression coefficient on the log scale.

**Table 6:** Differences in log-transformed trace metal concentrations between the two soft tissues (liver and kidney) based on the raw values.

Metal (mg/kg)	β	SE (HC3)	T	CI Lower er	CI Upper	*p*	exp(β)	*p*-Holm
Ti	−0.4610	0.0595	−7.7491	−0.5776	−0.3444	<0.01	0.6306	<0.001
V	0.1969	0.0770	2.5592	0.0461	0.3478	0.0105	1.2177	0.0315
Cr	−1.5240	0.0323	−47.1943	−1.5873	−1.4607	<0.001	0.2178	<0.001
Mn	−0.2029	0.0262	−7.7336	−0.2544	−0.1515	<0.001	0.8163	<0.001
Fe	−0.4960	0.0516	−9.6213	−0.5971	−0.3950	<0.001	0.6089	<0.001
Co	0.1123	0.0732	1.5345	−0.0311	0.2556	0.1249	1.1188	0.2498
Ni	−1.0546	0.0615	−17.1433	−1.1752	−0.9341	<0.001	0.3483	<0.001
Cu	−0.2902	0.0259	−11.1968	−0.3410	−0.2394	<0.001	0.7481	<0.001
Zn	−0.2962	0.0250	−11.8572	−0.3451	−0.2472	<0.001	0.7437	<0.001
As	0.6780	0.0543	12.4926	0.5717	0.7844	<0.001	1.9700	<0.001
Se	1.2414	0.0734	16.9204	1.0976	1.3852	<0.001	3.4604	<0.001
Mo	−0.7606	0.0242	−31.3995	−0.8081	−0.7131	<0.001	0.4674	<0.001
Sb	−0.5199	0.0653	−7.9576	−0.6480	−0.3919	<0.001	0.5946	<0.001
Cd	2.1961	0.1154	19.0296	1.9700	2.4223	<0.001	8.9903	<0.001
Hg	−0.1126	0.0746	−1.5090	−0.2589	0.0337	0.1313	0.8935	>0.09
Pb	−0.6269	0.0932	−6.7255	−0.8096	−0.4442	<0.001	0.5342	<0.001

Note. Two-sided *p*-values were adjusted using Holm–Bonferroni correction across the 17 tests. The concentrations of individual metals were modelled on the natural logarithmic scale as a function of organ type. Standard errors are heteroscedasticity-consistent (HC3) and clustered at the individual level. The coefficient β represents the unstandardized regression coefficient on the log scale. Estimated marginal means are reported.

**Table 7:** Differences in the bioaccumulation of trace metal concentrations between the two soft tissues (liver and kidney) using separate GAMMs with Holm-Bonferroni correction of *p*-values as sensitivity analysis.

Metal	Kidney Mean	Liver Mean	Beta	SE	z	*p*	*p* (Holm)	Liver/Kidney Ratio	95% CI for Ratio
Ti	0.126	0.187	0.399	0.065	6.181	<0.001	<0.001	1.490	1.313–1.691
V	0.013	0.010	−0.228	0.065	−3.518	<0.001	<0.001	0.796	0.701–0.904
Cr	0.016	0.071	1.511	0.050	30.072	<0.001	<0.001	4.529	4.104–4.998
Mn	2.086	2.566	0.207	0.023	8.823	<0.001	<0.001	1.230	1.175–1.288
Fe	85.311	140.655	0.500	0.036	13.918	<0.001	<0.001	1.649	1.537–1.769
Co	0.038	0.033	−0.138	0.031	−4.463	<0.001	<0.001	0.871	0.819–0.925
Ni	0.078	0.123	0.456	0.068	6.720	<0.001	<0.001	1.577	1.381–1.801
Cu	4.056	5.407	0.288	0.025	11.410	<0.001	<0.001	1.333	1.269–1.401
Zn	27.703	37.084	0.292	0.020	14.258	<0.001	<0.001	1.339	1.286–1.393
As	0.011	0.004	−0.989	0.049	−20.119	<0.001	<0.001	0.372	0.338–0.410
Se	1.124	0.330	−1.224	0.047	−25.872	<0.001	<0.001	0.294	0.268–0.322
Mo	0.488	1.041	0.759	0.022	34.699	<0.001	<0.001	2.135	2.046–2.229
Sb	0.002	0.004	0.549	0.087	6.304	<0.001	<0.001	1.732	1.460–2.055
Cd	0.908	0.100	−2.210	0.077	−28.798	<0.001	<0.001	0.110	0.094–0.127
Hg	0.015	0.011	−0.340	0.076	−4.472	<0.001	<0.001	0.712	0.613–0.826
Pb	0.044	0.082	0.632	0.057	11.056	<0.001	<0.001	1.881	1.682–2.104

Note. Data subject to analysis was based on LOD/2.

**Table 8:** Pairwise differences in log-transformed concentrations of 17 trace metals among sampling sites (Welch’s *t*-tests).

Metal	Region A	Region B	Mean Difference	SE	t	*p*	*p*-Holm
Ti	Forráskút	Rábapordány	−0.3974	0.1127	−3.5263	0.0006	0.0276
Ni	Forráskút	Jászberény	−0.5269	0.1490	−3.5374	0.0006	0.0276
		Lajta	−0.5735	0.1370	−4.1861	0.0001	0.0053
		Rábapordány	−0.7459	0.1391	−5.3608	<0.001	0.0410
Sb	Biharkeresztes	Forráskút	0.6384	0.1308	4.8804	<0.001	0.0410
		Rábapordány	0.5440	0.1248	4.3592	0.0001	0.0053
	Forráskút	Lajta	−0.2934	0.0878	−3.3426	0.0011	0.0410
Zn	Biharkeresztes	Lajta	0.1916	0.0617	3.1057	0.0033	0.0410
	Jászberény	Lajta	0.1893	0.0451	4.2012	0.0001	0.0053
	Lajta	Rábapordány	−0.1874	0.0427	−4.3847	<0.001	0.0410
Mn	Biharkeresztes	Jászberény	0.1516	0.0488	3.1058	0.0027	0.0410
		Lajta	0.1778	0.0434	4.0945	0.0001	0.0053
	Lajta	Rábapordány	−0.1384	0.0401	−3.4489	0.0008	0.0352
Cd	Biharkeresztes	Forráskút	0.8668	0.2575	3.3662	0.0011	0.0410
	Forráskút	Jászberény	−1.5151	0.3096	−4.8941	<0.001	0.0410
		Rábapordány	−0.8588	0.2740	−3.1341	0.0022	0.0410
	Jászberény	Lajta	1.1121	0.2788	3.9896	0.0002	0.0096
V	Biharkeresztes	Forráskút	1.1210	0.0825	13.5798	<0.001	0.0410
	Forráskút	Jászberény	−0.9946	0.1110	−8.9625	<0.001	0.0410
		Lajta	−1.0133	0.0614	−16.5062	<0.001	0.0410
		Rábapordány	−1.0890	0.0918	−11.8637	<0.001	0.0410
As	Biharkeresztes	Forráskút	0.3002	0.0943	3.1848	0.0021	0.0410
		Jászberény	0.3917	0.1059	3.6994	0.0004	0.0188
	Forráskút	Lajta	−0.5109	0.0786	−6.4989	<0.001	0.0410
	Jászberény	Lajta	−0.6025	0.0922	−6.5313	<0.001	0.0410
	Lajta	Rábapordány	0.3374	0.1000	3.3722	<0.001	0.0410
Co	Biharkeresztes	Forráskút	0.9711	0.0972	9.9865	<0.001	0.0410
		Jászberény	0.4566	0.1104	4.1375	<0.001	0.0410
	Forráskút	Jászberény	−0.5145	0.1203	−4.2768	<0.001	0.0410
		Lajta	−1.0864	0.0831	−13.0678	<0.001	0.0410
		Rábapordány	−0.6768	0.1175	−5.7587	<0.001	0.0410
	Jászberény	Lajta	−0.5720	0.0982	−5.8269	<0.001	0.0410
	Lajta	Rábapordány	0.4097	0.0948	4.3232	0.0001	0.0053
Hg	Biharkeresztes	Forráskút	−0.4805	0.1057	−4.5454	<0.001	0.0410
		Jászberény	−0.3689	0.1181	−3.1220	0.0026	0.0410
		Lajta	0.4062	0.0900	4.5142	<0.001	0.0410
	Forráskút	Lajta	0.8867	0.0872	10.1692	<0.001	0.0410
		Rábapordány	0.7634	0.1142	6.6861	<0.001	0.0410
	Jászberény	Lajta	0.7751	0.1019	7.6053	<0.001	0.0410
		Rábapordány	0.6517	0.1258	5.1818	<0.001	0.0410
Pb	Biharkeresztes	Lajta	−0.8683	0.1364	−6.3647	<0.001	0.0410
	Forráskút	Jászberény	−0.4302	0.1236	−3.4818	0.0009	0.0378
		Lajta	−1.0574	0.0982	−10.7638	<0.001	0.0410
	Jászberény	Lajta	−0.6272	0.1230	−5.0996	<0.001	0.0410
		Rábapordány	0.6297	0.1532	4.1103	<0.001	0.0410
	Lajta	Rábapordány	1.2569	0.1336	9.4063	<0.001	0.0410
Se	Biharkeresztes	Jászberény	−0.3030	0.0863	−3.5090	0.0008	0.0352
		Lajta	1.0602	0.1107	9.5763	<0.001	0.0410
		Rábapordány	0.4080	0.1255	3.2510	0.0016	0.0410
	Forráskút	Jászberény	−0.4821	0.0952	−5.0619	<0.001	0.0410
		Lajta	0.8810	0.1178	7.4800	<0.001	0.0410
	Jászberény	Lajta	1.3631	0.0980	13.9036	<0.001	0.0410
		Rábapordány	0.7110	0.1145	6.2102	<0.001	0.0410
	Lajta	Rábapordány	−0.6522	0.1338	−4.8732	<0.001	0.0410

Note. Each row represents a Welch’s *t*-test between two regions for a given trace metal. Values are based on natural logarithm–transformed concentrations. Holm–Bonferroni correction (two-sided) was applied separately for each metal. The significance level was set at α = 0.05. Estimated marginal means are reported.

## Data Availability

The data presented in this study are available on request from the corresponding author.
